# Evaluation of Laparoscopic Sleeve Gastrectomy With a Natural Band (Sewefy Wrap): Results From a Prospective, Randomized Controlled Trial

**DOI:** 10.7759/cureus.83733

**Published:** 2025-05-08

**Authors:** Alaa Sewefy, Taha H Kayed, Tamer E Esmaeel

**Affiliations:** 1 Surgery, Minia University Hospital, Minia, EGY; 2 Radiology, Minia University Hospital, Minia, EGY

**Keywords:** banded gastric bypass, banded sleeve gastrectomy, complications of banded bariatric procedures, laparoscopic sleeve gastrectomy, omental flap, teres ligament flap, weight regain

## Abstract

Background

Laparoscopic sleeve gastrectomy (LSG) is the most popular bariatric surgery procedure. Adding a synthetic band to LSG is reported to decrease the incidence of weight regain. However, the synthetic material has many drawbacks. This study aimed to evaluate the use of a natural flap as a band with LSG.

Methods

This study was a prospective, randomized controlled trial and included 80 patients with morbid obesity. It was conducted in the Minia University Hospital between November 2022 and February 2024. Participants were equally and randomly assigned to two groups. In one group, the patients underwent LSG and banding using either the Teres ligament (the round ligament of the liver) or a tight omental flap. In the second group, the standard LSG procedure was carried out without any band.

Results

The mean follow-up period was 13.5 months. At one year, the mean gastric volume was significantly less in the banded group (110.7 ml) than in the non-banded group (147.3 ml; p=0.000), and the mean % excess weight loss (EWL) was significantly higher in former (85.8%) than in the latter group (79.5; p=0.002). Similarly, the mean % total weight loss (TWL) was also significantly higher in the banded group (41.3%) than in the non-banded group (36.7%; p=0.010). Moreover, the mean score of food tolerance was 20.8 in the banded group versus 21.1 in the non-banded one (p=0.172).

Conclusions

LSG along with the a natural band using a nearby flap like the round ligament of the liver or an omental flap leads to better weight loss than non-banded LSG. It is also associated with minimal pouch dilatation and a low complication rate. The use of this technique is, hence, a promising procedure that minimizes sleeve pouch dilatation (the main cause of weight regain after LSG) without any extra cost or complications associated with a foreign body. However, long-term follow-up for this procedure is needed.

## Introduction

Obesity has reached epidemic proportions worldwide, accounting for more than 1.9 billion overweight and approximately 650 million obese adults. There is greater morbidity and mortality in patients with severe obesity, especially classes II (BMI 35.0 to 39.9) and III (BMI greater than 40). In addition, obesity is a recognized risk factor for the development of comorbid conditions such as cardiovascular disease, type 2 diabetes mellitus, malignancy, asthma, osteoarthritis, chronic back pain, obstructive sleep apnea, non-alcoholic fatty liver disease, and gallbladder disease [1-3].

Laparoscopic sleeve gastrectomy (LSG) is one of the most commonly performed bariatric surgeries worldwide. Although it is as effective as bariatric and metabolic surgery, weight regain remains an important concern. It is reported that there is a dilation of the gastric pouch over time, thus decreasing the long-term restrictive effect of the LSG. This phenomenon is common for super obese patients, and they usually need revision surgery for weight regain three to five years after the primary operation [4-7].

To prevent gastric pouch dilation and add more restrictions to the sleeved stomach, some authors proposed an additional restriction by placing a ring or band around the gastric tube to increase the long-term effect on weight loss. The silastic ring was used to prevent gastric pouch dilation in the Roux-en-Y gastric bypass (RYGB) and recently in LSG. Banded LSG was reported to counteract the sleeve pouch dilatation and minimize weight regain over time. The resulting weight loss was encouraging. However, many problems were reported with the synthetic bands, as evidenced by the laparoscopic adjustable gastric banding (LAGB), such as gastric band erosion, food intolerance, band slippage, and gut obstruction. Consequently, the popularity of LAGB declined. In the case of banded bariatric procedures, stenosis, erosions, and ring slippage are well-known complications in up to 7% of the patients [8-11].

Our study aims to test the efficacy of banding the sleeved stomach using a nearby flap (round ligament of the liver or an omental flap) as a natural band to prevent pouch dilatation and, at the same time, to avoid the complications observed with synthetic rings. The usage of the omental flap is common, especially in reconstructive surgery. Also, the Teres ligament flap has been reportedly used for cardiopexy of the hiatus hernia [12,13].

## Materials and methods

Study design

This prospective randomized controlled trial (registration ID: NCT05603338) included patients eligible for LSG. The study was conducted at the Minia University Hospital between November 2022 and February 2024. All patients completed at least one year of follow-up. The protocol was approved by the Faculty of Medicine, Minia University Ethical Committee (approval number 1062/11/2022). In the original protocol, the method detailed the use of an omental flap as a natural band. At the beginning of the study, five patients were operated on using a tight omental flap as a natural flap along with LSG. We then shifted to using the round ligament of the liver as a natural band, as it was much stronger. If it was very short, we used a tight omental flap. This amendment to the methods was approved by the ethical committee. The study has been reported in line with Consolidated Standards of Reporting Trials (CONSORT) Guidelines [14].

Participants

Patients were included in the study if they were >18 and <65 years old and had a BMI of more than 35 without comorbidities or more than 30 with comorbidities. We excluded patients who had previous bariatric procedures, patients with preoperative manifestations of gastroesophageal reflux disease (GERD), pregnancy, inflammatory bowel disease, and patients who refused to participate in the study.

Randomization and blinding

Method of Randomization

The allocation sequence was generated using a permuted block randomization technique, and the block size was variable. The allocation sequence/code was concealed from the person allocating the participants to the intervention arms using sealed opaque envelopes.

Blinding

The participants, radiologist, outcome assessors, and the data analyzer were blinded to group allocation of the patients.

Intervention

**Figure 1:**
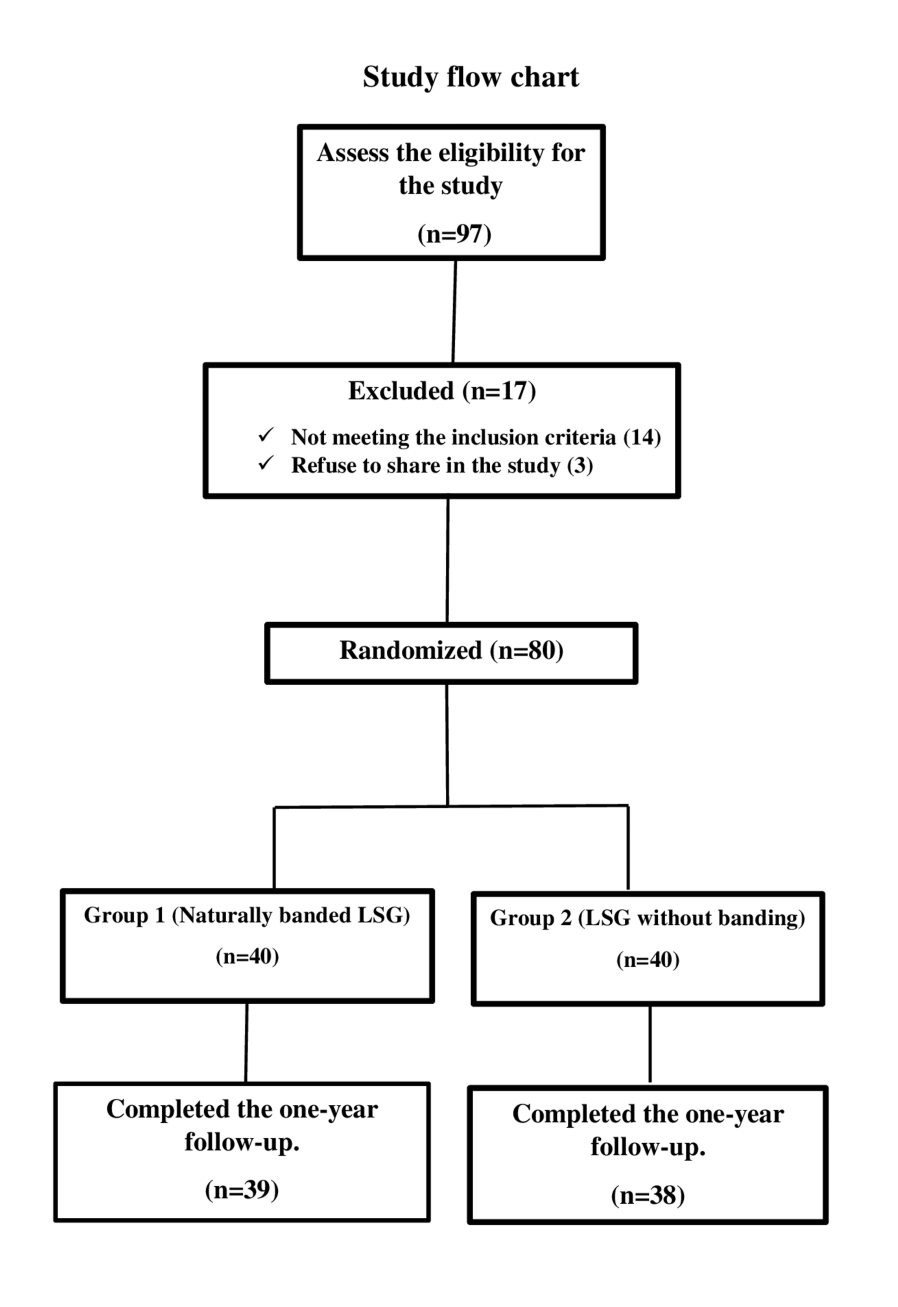
Study flow chart

The 40 patients in the banded group underwent LSG plus banding using a natural flap (round ligament of the liver or a tight omental flap). On the other hand, the 40 participant in the non-banded group were operated via standard LSG without banding (Figure 1).

All the patients were invited to participate in this study and the details about the risks and the benefits of each procedure were explained. All the included participants gave their written informed consent. All the procedures were done in accordance with the ethical standards of the 1964 Helsinki Declaration and its later amendments.

Study outcomes

The primary outcome was the weight loss parameters at one year and included:

Percentage of excess weight loss (%EWL) at one year after surgery. %EWL was measured as (initial weight - follow-up weight)/(initial weight - bodyweight at a BMI of 25 kg/ m^2^) x 100 [15].

Percentage of total weight loss (%TWL) at one year, calculated as: (preoperative weight - follow up weight)/preoperative weight) × 100 [15].

Secondary outcomes included:

Gastric pouch size at one year, estimated by gastric volumetry using multi-detector computed tomography (MDCT).

Operative time, measured from skin incision to skin closure [15].

Complications, according to the Clavien-Dindo classification [16].

Food tolerance, assessed using an Arabic questionnaire to assess the patient's overall tolerance to food, with a score ranging from one to 27 one year after surgery, with 27 being the maximum for excellent food tolerance [17].

Study endpoints

Weight loss, gastric volume, food tolerance score, and the incidence of complications (especially new GERD) at one year.

Preoperative care

A careful evaluation of the medical history, complete physical examination, and necessary investigations such as complete blood picture (CBC), liver and renal functions, HbA1C, random blood sugar, electrocardiogram, and respiratory function test (if indicated) were carried out. Upper gastrointestinal tract (GIT) endoscopic examination was carried out in patients that had symptoms of GERD, and anticoagulant prophylaxis was administered 12 h before the operation [15]. 

Surgical procedure

As is standard for LSG, the patients were operated in the French position with a steep reverse-Trendelenburg. The five ports technique was used. The first step was to divide the greater omentum from the stomach upward to the left crus of the diaphragm and down near the pylorus. The crus were cleared from any adhesion. Any posterior adhesions to the stomach were completely removed. We began cutting the stomach at 2-4 cm from the pylorus. After complete division the stomach, the staple line was routinely plicated using a running 3/0 Prolene suture (Ethicon, New Jersey, US). The sleeved stomach was anchored to the diaphragm to prevent postoperative migration into the thoracic cavity. Additionally, it was affixed to the peripancreatic fascia and, at its distal third, to the omentum to minimize the risk of twisting [15].

**Figure 2:**
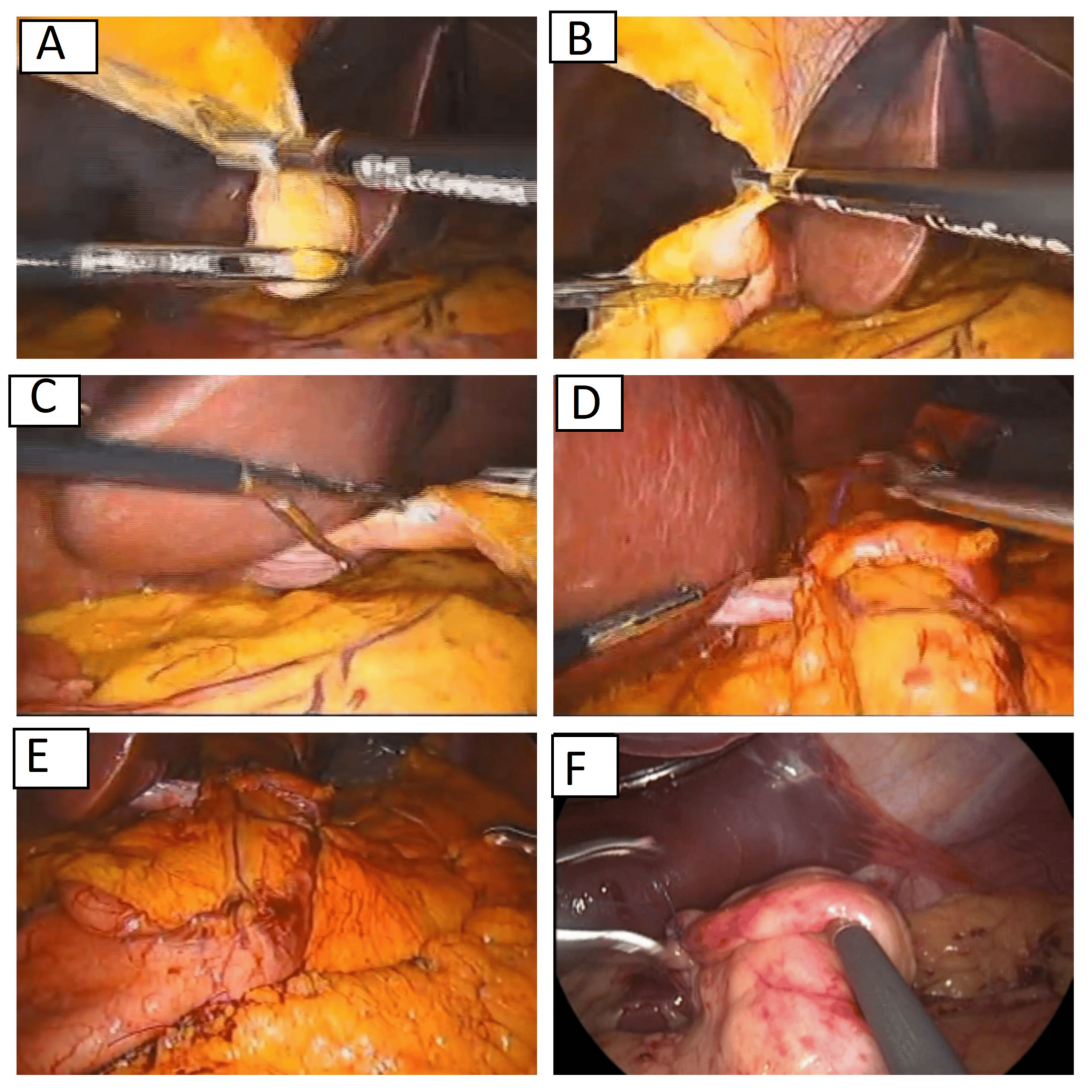
Technique for the laparoscopic sleeve gastrectomy using a natural band A, B, C: Dissection of the round ligament. D: Passing the ligament around the stomach and suturing it to itself. E & F: The final appearance.

In banded group, the round ligament of the liver was first evaluated. If it was long enough to be wrapped around the sleeved stomach, it was separated from the umbilicus and dissected distally to the maximum extent possible while preserving its attachment to the point of origin. The pars flacida was then opened. The dissected round ligament was positioned around the sleeved stomach at 4-5 cm below the esophagogastric junction to form of a loop. It was then sutured to itself and to the sleeved stomach, encasing a 36 French calibrating tube (MID-TUBE, Arabian Co., Egypt), to function as a constrictive band (Figure 2, Video 1).

**Video 1:** Technique for the laparoscopic sleeve gastrectomy using a natural band

If the round ligament was too short to pass around the stomach, a dissected omental flap was prepared and was wrapped tightly around the sleeved stomach 4-5 cm below the esophagogastric junction, with the 36 French calibrating tube in place (video 1). The same senior consultant of bariatric surgery operated on all the patients.

Evaluation with gastric voltammetry

In all the patients, the sleeve volume was evaluated using an MDCT virtual gastroscopy one month and one year after surgery. MDCT gastrographic volumetry was performed with a 64-detector multi-detector CT scanner (Canon Aquilion Prime SP®, Canon Medical Systems, USA). Patients were asked to fast for at least six hours before the procedure and given an intravenous injection of 40 mg butylscopolamine. They were then asked to swallow two to four packs of sodium bicarbonate granules, as tolerated. The same consultant radiologist did all the gastric volumetric studies.

Follow-up

Early ambulation was encouraged. Five hours after the surgery, clear fluids were allowed. Thromboprophylaxis was prescribed for two weeks and proton pump inhibitors (PPIs) for three months. Follow-up visits occurred once a week for one month, and then once a month. The follow-up was carried out by clinic visits, regular telephone calls, and WhatsApp messages, which included a Google form (Google, California, US). Social media platforms and remote follow-up were reported to be safe and effective. There was a follow up timetable that was calendarized for all the patients and if any patient was delayed, they received a follow-up call and or a message from the secretary of the project. If a patient developed any complaint at any time, they could visit the clinic. The patients followed a regimen of oral fluids for the first two weeks, followed by a soft diet during the third week, and then gradually low-calorie, high-protein food was added to their diet. A high-concentration multivitamin supplement was also prescribed after the third week [15,18,19].

Sample size calculation

The sample size was calculated using G*power software (version 3.1.9.7 Heinrich-Heine-Universität Düsseldorf, Düsseldorf, Germany) based on previous studies that compared banded LSG to non-banded LSG, that were published till the time of protocol writing. We found that the mean %EWL at one year in banded LSG varies from 52-77% with an average of 64.2% and %EWL at one year in non-banded LSG varies from 41-61% with average of 54%. The average SD in both groups was 15.6 and 14.9, respectively (using independent t-test, means: different between two independent means/groups, 2 tails, equal allocation and alpha of 0.05), and the effect size was 0.668 with a power of 80%. The minimal sample needed for the study was estimated to be 74 patients, 37 in each group. We suspected up to a 10% drop off, so 40 patients were included in each group [9,20-23].

Data collection

Baseline data collected included sex, age, body height, and weight, BMI, the presence of gallstones, and obesity-related comorbidities. The operative time and early postoperative complications were also noted. Additionally, the size of the gastric pouch at one month and at the end of the first year were evaluated. At the end of one year, late postoperative complications, improvement of comorbidities, food tolerance, and the %EWL were assessed. 

Statistical analysis

The IBM SPSS Statistics for Windows, Version 25 (Released 2017; IBM Corp., Armonk, New York, United States) was used to collect and analyze data. The continuous variables proved to be normally distributed by the Shapiro-Wilk test. So parametric statistics was adopted. Quantitative data were presented by mean and SD, while qualitative data were presented by frequency distribution. The student's t-test was used to compare continuous variables, and the Chi-square test was used to compare categorical or ordinal variables. An alpha level was set to 5% with a significant level of 95%. Statistical significance was tested at p-value <0.05.

## Results

Baseline data

This study included 80 patients who underwent LSG. Fifty-five patients (68.8%) were female, and 25 (31.2%) were male. The mean BMI was 47.9 kg/m^2^, and the mean age was 36.3 years. With respect to comorbidities, diabetes mellitus (DM) was present in nine (11.3%), hypertension in 21 (26.3%), gallstones in seven (8.8%), obstructive sleep apnea syndrome (OSAS) in 15 (18.8%), and hyperlipidemia in 45 (56.3%) patients with no significant difference in the preoperative data between the two treatment groups. The mean period of follow-up was 13.5 months with a minimum of 12 months and a maximum of 16 months (Table 1).

**Table 1:** Preoperative, postoperative, and follow-up data of the patients (n=80) Quantitative data were presented by mean and standard deviation, while qualitative data were presented by frequency distribution. Student's t-test was used to compare continuous variables, and the Chi-square test was used to compare categorical or ordinal variables. An alpha level was set to 5% with a significance level of 95%. Statistical significance was tested at p-value <0.05. *Teres ligament (n=33) and Omentum (n=7); DM, Diabetes Mellitus; HPN, Hypertension; OSAS, Obstructive sleep apnea syndrome

	Total (n=80)	Banded group* (n=40)	Non-banded group (n=40)	P-value
Age (years)	36.3±12.4	36.6±12.50	36±12.5	0.42
BMI (kg/m^2^)	47.9±5.7	47.4±5.8	47.1±7.6	0.723
Female	55 (68.8%)	27 (67.5%)	28 (70%)	0.809
Male	25 (31.2%)	13 (32.5%)	12 (30%)	
Gallstone	7 (8.8%)	3 (7.5%)	4 (10%)	0.692
DM	9 (11.3%)	5 (12.5%)	4 (10%)	0.723
HPN	21 (26.3%)	10 (25%)	11 (27.5%)	0.799
Hyperlipidemia	45 (56.3%)	21 (52.5%)	24 (60%)	0.499
OSAS	15 (18.8%)	8 (20%)	7 (17.5%)	0.775
Operative time (minutes)		37±4	33±5	<0.001
Follow-up period (months)	13.5±1	13.9±1.1	13.7±1.1	0.402

Preoperative gallstones were present in seven (8.8%) patients. They were all managed by cholecystectomy in the same session without any complications. The mean operative time was 37±4 minutes in the banded group vs. 33±5 minutes in the non-banded group (p<0.001). In the banded group, the sleeved stomach was banded using a tight omental flap in seven patients and the Teres ligament in was used in 33 patients (Table 1).

Weight loss

**Figure 3:**
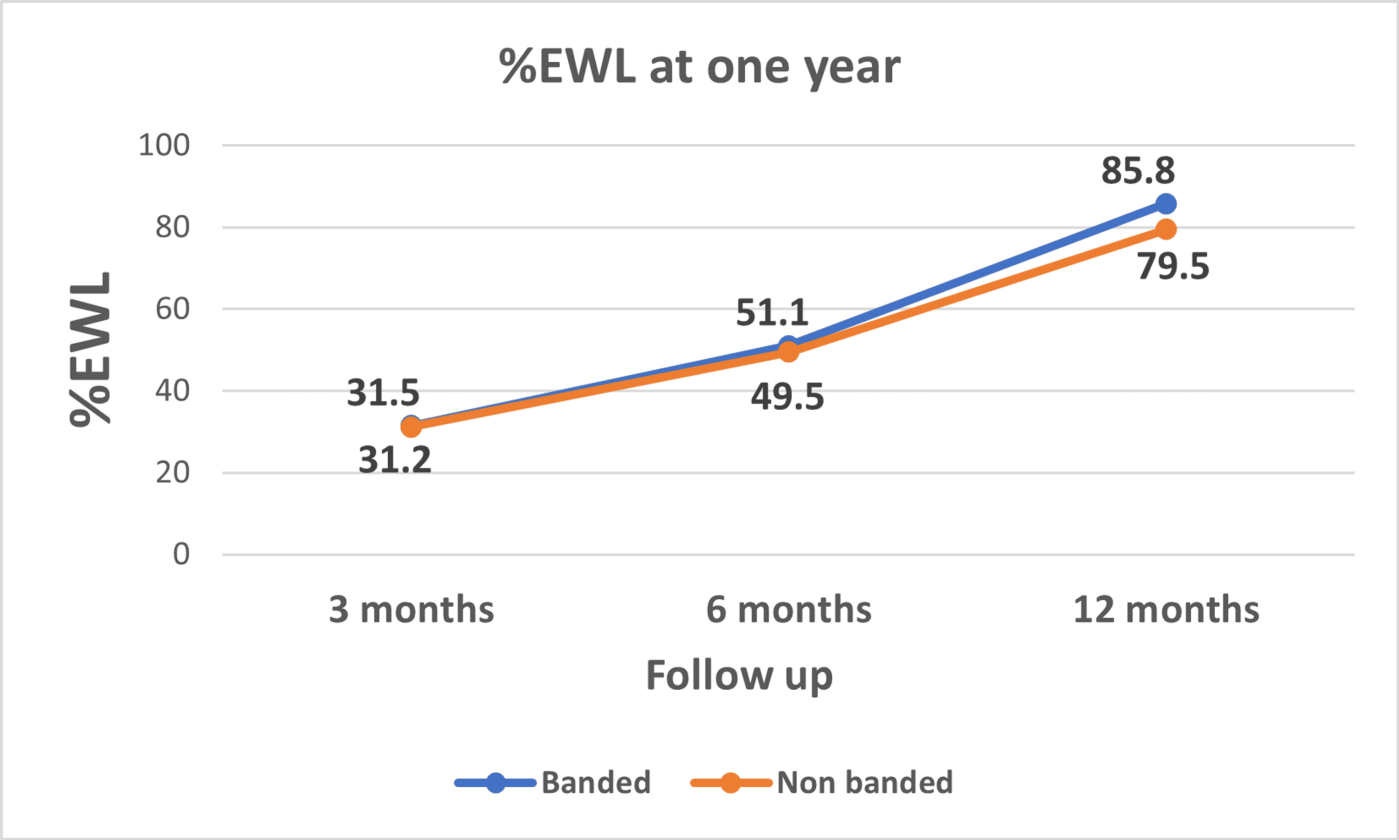
Percentage of excess weight loss (%EWL) over 12 months

**Figure 4:**
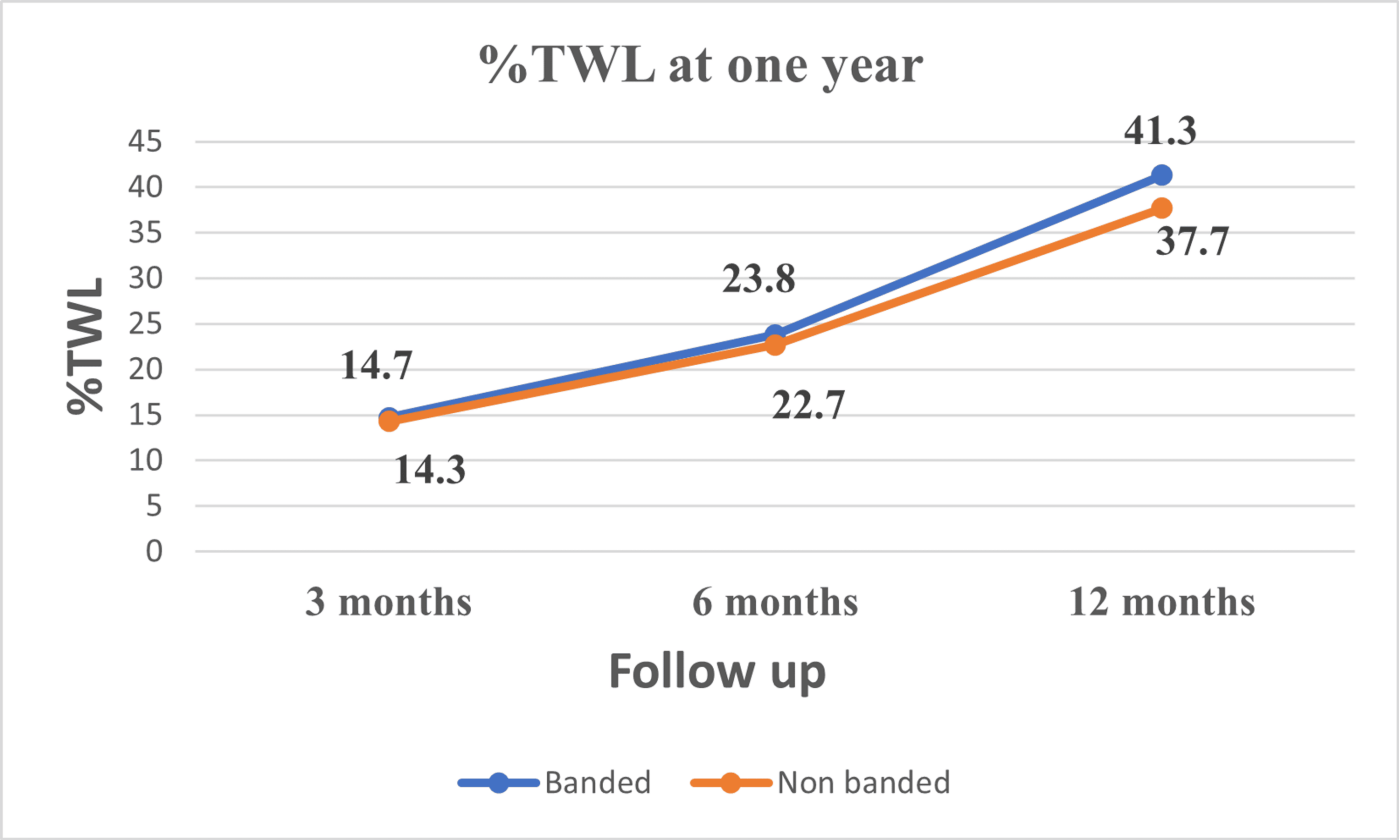
Percentage of total weight loss (%TWL) over 12 months

There was no significant difference in the weight loss parameters between the two groups at three and six months after the operation. At one year, %EWL was 85.8% ± 8.7 in banded group vs. 79.5% ±8.4 in the non-banded group (p=0.002). Similarly, %TWL at one year was 41.3% ±7.5 in the banded group vs. 36.7% ±7.6 in non-banded group (p=0.010). (Table 2, Figures 3, 4).

**Table 2:** Weight loss parameters and food tolerance over 12 months in the banded and non-banded groups Data were presented as mean and standard deviation. Student's t-test was used to compare variables. Statistical significance was tested at p-value <0.05. %EWL, percentage of excess weight loss; BMI, Body mass index; %TWL, percentage of total weight loss

Variable		Group		P-value
		Banded (n=39)	Non-banded (n =38)	
%EWL	At 3 months	31.5±5	31.2±6	0.728
	At 6 months	51.1±6.8	49.5±6.1	0.283
	At 12 months	85.8±8.7	79.5±8.4	0.002
%TWL	At 3 months	14.7±2.3	14.3±2.4	0.402
	At 6 months	23.8±3.1	22.7±3.1	0.140
	At 12 months	41.3±7.5	36.7±7.60	0.010
Food tolerance score	At 3 months	10.4±1.1	12.5±2	0.000
	At 6 months	15.1±1.6	17.2±1.1	0.000
	At 12 months	20.8±.9	21.1±1	0.172

Gastric volume

There was no significant difference between the two treatment groups with respect to the gastric volume at one month (96±3.2 ml and 95.2±5.7 ml for the banded and non-banded groups, respectively). The mean gastric volume at one year was significantly less in the banded group (110.7±4.5 ml) than in the non-banded group (147.3±10.3 ml; p=0.000) (Table 3). 

**Table 3:** Change in gastric volume at one year in the banded and non-banded groups Data were presented by mean and standard deviation. Student's t-test was used to compare variables. Statistical significance was tested at p-value <0.05.

	Banded group (n=39)	Non-banded group (n=38)	P-value
At one month (ml)	96±3.2	95.2±5.7	0.47
At 12 months (ml)	110.7±4.5	147.3±10.3	0.000
Change in gastric volume (ml)	16.7±4.8	47.1±12.1	0.000

Complications

In the banded group, one patient (2.6%) developed intramural bleeding on the second postoperative day, and presented with hematemesis and melena. The patient's condition improved with conservative management. In the non-banded group, one patient (2.6%) developed deep vein thrombosis (DVT) in the second week after the procedure and was managed by therapeutic anticoagulation. One (2.6%) and two (5.3%) patients developed anemia in the banded group and non-banded groups, respectively (p=0.541). Vitamin D deficiency developed in three patients (7.7%) in the banded group compared to one patient (2.6%) in the non-banded group (p=0.317), and de novo GERD occurred in five patients (12.8%) in the former group vs. three patients (7.9%) in the latter (p=0.479). Moreover, one patient in the banded group needed conversion to RYGB due to intractable GERD. Diagnosis of GERD was based on the clinical manifestations and by endoscopic examination (Table 4).

**Table 4:** Complications in the banded and non-banded groups Data were presented by frequency distribution. Chi-square test was used to compare variables. Statistical significance was tested at p-value <0.05. *Complications according to the Clavien-Dindo classification [16]. DVT, Deep venous thrombosis; GERD, gastroesophageal reflux disease

Type of complications*		Banded group (n=39)	Non-banded group (n=38)	Grade	P-value
Bleeding		1 (2.6%)	0%	Grade II	0.32
DVT		0	1 (2.6%)	Grade II	0.32
Nutritional	Anemia	1 (2.6%)	2 (5.3%)	Grade II	0.541
	Vitamin D deficiency	3 (7.7%)	1 (2.6%)	Grade II	0.317
de novo GERD		5 (12.8%)	3 (7.9%)	Grade II-Grade III	0.479

Food tolerance

The score for food tolerance was significantly lower in the banded group compared to the non-banded group at three and six months but not at one year after the procedure. The mean score of food tolerance at one year was 20.8±0.9 in the former group vs. 21.1±0.1 in the latter (p=0.172) (Table 2, Figure 5).

Remission of the comorbidities

Remission of the comorbidities varied across both groups at one year. The number of patients with DM reduced by 80% in the banded group vs. 75% in the non-banded group (p=0.858). On the other hand, patients with hypertension decreased by 70% in the former group vs. 72.7% in the latter (p=0.890). Similarly, there was a 75% and 71.4% reduction in patients with OSAS in the banded and non-banded groups, respectively (p=0.876). Lastly, patients with hyperlipidemia reduced by 61.9% and 70.8% in the former and latter groups, respectively (p=0.526) (Table 5). 

**Table 5:** Remission of the comorbidities at one year in the banded and non-banded groups Data were presented by frequency distribution.  Chi-square test was used to compare variables. Statistical significance was tested at p-value <0.05. DM, Diabetes Mellitus; HPN, Hypertension; OSAS, Obstructive sleep apnea syndrome; HLP, Hyperlipidemia

	Banded group (n=39)			Non-banded group (n=38)			P-value
	Before procedure	Post procedure	% of remission	Before procedure	Post procedure	% of remission	
DM	5	1	80%	4	1	75%	0.858
HPN	10	3	70%	11	3	72.7%	0.890
OSAS	8	2	75%	7	2	71.4%	0.876
HLP	21	8	61.9%	24	7	70.8%	0.526

## Discussion

As mentioned before, the main drawback of LSG is pouch dilatation, which leads to weight regain over time. Using MDCT for a 3D gastric volumetric study is now considered the best method to analyze the shape/volume of the gastric pouch in bariatric surgery [24,25]. In this study, the mean gastric volume one month after the procedure was 96±3.2 ml and 95±5.7 ml in the banded and non-banded groups, respectively. In contrast, the mean gastric volume at one year was 110.7±4.5 ml in the banded group versus 147.3±10.3 ml in the non-banded group, with statistically significant pouch dilatation in the latter group. Ali et al. reported that the gastric volume at one month after non-banded LSG ranged from 60-107 ml with a median of 82.9 ml, while the mean gastric volume at one year was 171.6 ml. Thus, there was a significant increase in the gastric pouch volume after one year [24]. On the other hand, Hany et al. reported a mean gastric volume of 177.6 ml in non-banded LSG vs. 111 ml in banded LSG at one year [10]. Braghetto et al. found that the early postoperative gastric volume in non-banded LSG was 116 ml and it increased to 254 ml after two years [25]. Our initial sleeve volume was slightly less than that reported in other studies due to many technical factors, as we used 36 French bougie, routinely plicated the stable line, and finally, carried out more antral resection.

In this study, the %EWL and %TWL at one year in the banded group (85.8% and 41.3%, respectively) were significantly better than those in the non-banded group (79.5% and 36.3%, respectively). Most studies that compared banded LSG (using the synthetic ring) with non-banded LSG reported a significantly better weight loss in the former than the latter group, especially in the midterm and the long-term results [8,10,20,26-29]. The reported %EWL in literature for banded LSG at one year ranged from 52-77%. However, in the non-banded groups, it ranged from 41-61%. Our result is higher than that reported in the literature, which may be due to the smaller gastric pouch caused by the small bougie size and more antral resection, which reportedly improves weight loss [30-32].

The operative time in this study was 37 minutes in the banded group vs. 33 minutes in the non-banded group. It is less than that reported in the literature, which ranges from 49-87 minutes and 46-75 minutes, respectively. This is because all patients in this study were operated on by the same expert surgeon with extensive experience. It is well established that increased surgical expertise is associated with reduced operative time. For instance, Carandina et al. reported a 40-minute decrease in operative duration between the early and later phases of surgical experience with LSG [33-35].

Food tolerance is a major problem in banded LSG. In this study, there was a significantly lower food tolerance score in the banded group compared to the non-banded group at three and six months of follow up. But the mean score of food tolerance at one year was similar with 20.8 in the banded group vs. 21.1 in the non-banded group. Hany et al. reported a mean score of food tolerance of 21.46 in banded LSG versus 21.43 in non-banded LSG at one year [10]. However, many studies have reported that food intolerance in the form of vomiting, regurgitation, and dysphagia is more common in the former than in the latter groups [21,23,26,28].

In this study, de novo GERD occurred in 12.8% of patients in the banded group vs. 7.9% in the non-banded group. In the systematic reviews and meta-analysis by Chaouch et al., the authors reported a rate of 13.8% and 19.6% in the banded and non-banded LSG groups [4]. On the other hand, Hany et al. found the incidence of de novo GERD to be 20.2% in the non-banded vs. 18.9% in the banded LSG group [10].

Study limitations

Our study compared naturally-banded LSG with non-banded LSG, and it would have been better if it had included a third group which evaluated banded LSG using a synthetic ring. The two banding methods could then have been compared regarding their impact on weight loss and the incidence of complications. In the banded group, seven patients were operated using the omental flap as a band, and it was better to divide the banded group to two equal subgroups and compare the results between these two subgroups. Since all the patients were operated on by the same surgeon, the results may not be applicable to procedures performed by other surgeons. Moreover, it was a short-term study, hence more long-term evaluation is needed to confirm the results.

## Conclusions

Naturally banded LSG using a nearby flap like the round ligament of the liver or an omental flap, leads to better weight loss than non-banded LSG. It is associated with minimal pouch dilatation, low complications rate, so the use of this technique is a promising procedure that minimizes the sleeve pouch dilatation (the main cause of weight regain after LSG) without an extra-cost or foreign body complications but, long-term follow-up is needed
